# Effects of perineal massage at different stages on perineal and postpartum pelvic floor function in primiparous women: a systematic review and meta-analysis

**DOI:** 10.1186/s12884-024-06586-w

**Published:** 2024-06-03

**Authors:** Jinzhu Yin, Yun Chen, Meiling Huang, Zhongyan Cao, Ziyan Jiang, Yao Li

**Affiliations:** 1 The Third Clinical College of Guangzhou Medical University, The Nursing College of Guangzhou Medical University, Guangzhou, China; 2 Department of Nursing, Guangdong Provincial Key Laboratory of Major Obstetric Diseases, Guangdong Provincial Clinical Research Center for Obstetrics and Gynecology, The Third Affiliated Hospital of Guangzhou Medical University, Guangzhou, China; 3 Department of Obstetrics and Gynecology, Obstetrics; Guangdong-Hong Kong-Macao Greater Bay Area Higher Education Joint Laboratory of Maternal-Fetal Medicine, The Third Affiliated Hospital of Guangzhou Medical University, Guangzhou, China

**Keywords:** Perineal massage, Primiparous women, Antenatal, Second stage of labor, Perineum, Pelvic floor

## Abstract

**Background:**

Perineal massage, as a preventive intervention, has been shown to reduce the risk of perineal injuries and may have a positive impact on pelvic floor function in the early postpartum period. However, there is still debate concerning the best period to apply perineal massage, which is either antenatal or in the second stage of labor, as well as its safety and effectiveness. Meta-analysis was used to evaluate the effect of implementing perineal massage in antenatal versus the second stage of labor on the prevention of perineal injuries during labor and early postpartum pelvic floor function in primiparous women.

**Methods:**

We searched nine different electronic databases from inception to April 16, 2024. The randomized controlled trials (RCTs) we included assessed the effects of antenatal and second-stage labor perineal massage in primiparous women. All data were analyzed with Revman 5.3, Stata Statistical Software, and Risk of Bias 2 was used to assess the risk of bias. Subgroup analyses were performed based on the different periods of perineal massage. The primary outcomes were the incidence of perineal integrity and perineal injury. Secondary outcomes were perineal pain, duration of the second stage of labor, postpartum hemorrhage, urinary incontinence, fecal incontinence, and flatus incontinence.

**Results:**

This review comprised a total of 10 studies that covered 1057 primigravid women. The results of the analysis showed that perineal massage during the second stage of labor reduced the perineal pain of primigravid women in the immediate postpartum period compared to the antenatal period, with a statistical value of (MD = -2.29, 95% CI [-2.53, -2.05], *P* < 0.001). Additionally, only the antenatal stage reported that perineal massage reduced fecal incontinence (*P* = 0.04) and flatus incontinence (*P* = 0.01) in primiparous women at three months postpartum, but had no significant effect on urinary incontinence in primiparous women at three months postpartum (*P* = 0.80).

**Conclusions:**

Reducing perineal injuries in primiparous women can be achieved by providing perineal massage both antenatally and during the second stage of labor. Pelvic floor function is improved in the postnatal phase by perineal massage during the antenatal stage.

**Trial registration:**

CRD42023415996 (PROSPERO).

**Supplementary Information:**

The online version contains supplementary material available at 10.1186/s12884-024-06586-w.

## Introduction

One of the most typical postpartum consequences of vaginal delivery is perineal trauma. According to statistics [1, 2], perineal injuries of varying degrees occur in 53–89% of women experiencing vaginal delivery, with the highest incidence in primiparous women [3], including spontaneous and human intervention-induced perineal injuries [4]. However, both short-term and long-term complications are linked to perineal trauma during childbirth [5, 6]. Hemostasis, bleeding, infection, and a delay in the mother-infant bonding process are short-term problems [5]. Perineal discomfort, weakening in the pelvic floor muscles, fecal or urinary incontinence, and a delayed return to regular sexual activity are long-term consequences [7].

Several preventive measures have been suggested to minimize complications during childbirth. One such measure is pelvic floor muscle training (PFMT) during pregnancy, which has been found to enhance the strength and flexibility of the pelvic floor muscles, thereby reducing the risk of perineal injury [8]. Early and structured PFMT can also help lower the occurrence of urinary incontinence in late pregnancy and the first six months after giving birth [9]. However, many women may struggle to maintain regular exercise and master the required techniques for PFMT. Specialized equipment like the Epi-No vaginal birth trainer can increase perineal tissue elasticity and reduce tearing and lateral incisions during labor, but this may come with additional costs and the need to purchase the equipment [10]. Birthing balls and free-position labor are believed to contribute to lower rates of perineal injuries [11, 12], although they require specific birthing settings and medical assistance. Additionally, physiotherapy techniques such as applying warm compresses to the perineum can enhance local blood circulation, but their effectiveness may be limited due to the need for quick application during delivery [13].

Apart from these procedures, perineal massage exhibits special benefits as an easy-to-use, inexpensive, and straightforward prophylactic measure. Perineal massage has been demonstrated in studies to increase perineal integrity in primiparous women [14], decrease perineal laceration and episiotomy, and promote perineal flexibility. Perineal massage dramatically reduces the risk of perineal injury in women who have given birth vaginally for the first time, according to Cochrane’s systematic evaluation [15]. Perineal massage has been recommended for antenatal use in certain studies [16, 17] and for use in the second stage of labor in others [18, 19]. A thorough meta-analysis of perineal massage techniques used at different periods is required to evaluate their effect on the health outcomes of primiparous women, as variable timing of intervention may result in diverse effects.

Prior systematic reviews [20, 21] have concentrated on the short-term effects of perineal massage for perineal injury, with comparatively less attention paid to examining the treatment’s long-term effects. Given the increasing amount of information available on perineal massage, it is necessary to conduct a comprehensive systematic review once again.

The objective of this systematic review and meta-analysis was to evaluate the effect of implementing perineal massage in antenatal versus the second stage of labor on the prevention of perineal injuries during labor and early postpartum pelvic floor function in primiparous women. Specifically, outcomes of the effects of perineal massage on the perineum include perineal integrity and perineal injury. Furthermore, this study will explore specific outcomes of the effects of perineal massage on pelvic floor function in the early postpartum period, such as urinary incontinence, fecal incontinence, and flatus incontinence.

## Methods

Following the Cochrane Handbook for Systematic Reviews of Interventions to the letter, we conducted this systematic review and meta-analysis [22]. The Preferred Reporting Item for Systematic Reviews and Meta-analyses (PRISMA) statement [23] was adhered to while reporting the meta-analysis.

### Literature search

We searched nine different electronic databases for randomized controlled trials examining the effects of perineal massage initiated at different stages on perineal and early postpartum pelvic floor function in primiparous women in the following databases: PubMed, Web of Science, Embase, Cochrane Library, CINHAL Plus, China National Knowledge Infrastructure (CNKI), Wanfang Database, China Science and Technology Journal Database (CQVIP), and China Biology Medicine (CBM) database. The search was conducted from the inception of the databases up to April 2023. The search was later updated on 16 April 2024. The search strategy was organized around the search terms “perineum” and “massage” and adapted to each database as needed. Searches were performed using a combination of MeSH terms, free text terms, and Boolean operators. As a supplement, manual searches were also performed. The PICOS (Population, Intervention, Comparison, Outcome, Study Design) framework was developed and used to guide the screening process. Supplementary file no. 1 contains the framework as well as the PubMed and CNKI search strategies. Reference lists of included studies and relevant reviews were screened to ensure that all appropriate studies were found. Language restrictions were applied for Chinese and English.

### Eligibility criteria

We included RCTs that met the following inclusion criteria: (1) population: primiparous women (age ≥ 18 years): singleton, cephalic presentation, estimated fetal weight less than 4000 g, no significant maternal complications or comorbidities during pregnancy and delivery; (2) intervention: perineal massage will be performed after 34 weeks of pregnancy or during the second stage of labor; (3) comparator: routine care; (4) outcome parameters: our primary outcomes include perineal integrity and perineal injury (including first- to fourth-degree perineal lacerations and episiotomy). Secondary outcomes include the duration of the second stage of labor (in hours or minutes), postpartum blood loss, perineal pain assessed using a visual analog scale (VAS), reported urinary incontinence, fecal incontinence, and flatus incontinence within three months postpartum; (5) study design: randomized controlled trials (RCTs).

We excluded studies for the following reasons: (1) Studies for which the full text was unavailable and the data could not be converted; (2) Combining additional interventions (such as Kegel exercises or perineal hot packs) with perineal massage in the experimental group; (3) Reviews, conference abstracts, etc.; (4) Studies with lower quality literature; (5) Non-English or Chinese literature. Two reviewers independently performed the title/abstract screening and full-text screening of the eligible studies. Differences were discussed, and a consensus was reached after the discussion.

### Study selection

The procedure of selecting studies consisted of four parts. First, a researcher imported the records retrieved from the database into EndnoteX9 and deleted duplicate records. This researcher imported the Endnote file into the second researcher’s computer after copying it and removing any duplicates to guarantee the impartiality of the study selection procedure. Then, using their individual computers, the two researchers separately went through the titles and abstracts, classifying each record as “included,” “uncertain,” or “excluded” according to the qualifying requirements. The entire text of the records designated as “included” and “uncertain” was then obtained by the researchers in order to evaluate eligibility. After thoroughly reviewing the entire literature, the researchers meticulously documented the studies that were excluded and provided a comprehensive description and classification of the reasons for their exclusion. Lastly, the researchers looked through the reference lists of the studies that were included, applying the “snowball method” to locate and include pertinent papers. Before examining the screening results, the two researchers had no communication. If any differences arose during the study screening procedure, the two researchers engaged in discussions and reached a consensus.

### Quality assessment

Two researchers independently assessed the quality of the trials using the most recent Cochrane risk of bias 2.0 methodology for randomized trials [24]. The Cochrane risk of bias 2.0 tool has been revised and now includes five assessment domains: “bias arising from the randomization process”, “bias due to deviations from intended interventions”, “bias due to missing outcome data”, “bias in measurement of the outcome” and “bias in selection of the reported result”. The risk of bias was classified as “low risk,” “some concerns,” or “high risk” for each assessment topic based on the responses to a series of “signaling questions” and the use of the risk of bias judgment pathway map. Subsequently, based on the individual evaluation outcomes for every domain, the overall bias of all the included studies was subsequently classified as either “low risk,” “some concerns,” or “high risk.” The “Excel tool with macros” was used to finish the quality assessment procedure. Disagreements that arose during the quality assessment process were resolved through consulting a third senior expert.

### Data extraction

Two authors (Jinzhu Yin & Zhongyan Cao) collected the data from eligible studies on a standardized data extraction sheet. We extracted the data, such as the following: author (publication year), mean age of participants, country, sample size, intervention details (such as performer, start time, technique, specific operation time, frequency, stop time, total duration, and auxiliary), comparison details, and outcome details.

### Data synthesis

All statistical analyses were performed via Review Manager Version 5.3 and Stata version 17.0. P-values and I^2^ values were used to measure heterogeneity first. An I^2^ < 50% and *P* > 0.1 in the absence of statistical heterogeneity indicated the application of a fixed-effects model. Sensitivity analysis was done to determine the probable sources of heterogeneity if statistical heterogeneity was observed (*P* < 0.1, I^2^ > 50%). A random-effects model was used in cases when heterogeneity could not be completely removed. Continuous variables of the data in this study were analyzed using mean difference (MD) if the same measurement tool was used or standardized mean difference (SMD) if a different measurement tool was used, and a 95% CI was calculated. Relative risks (RR) and matching 95% confidence intervals (CIs) were utilized for dichotomous variables. *P* < 0.05 was used as the statistical significance threshold. All included studies were divided into two subgroups: antenatal perineal massage and second stage of labor perineal massage, based on subgroup analyses based on various start periods of perineal massage. We performed sensitivity analyses with RevMan 5.3. Sensitivity was analyzed using the leave-one-out method, aiming to assess the susceptibility of the results of this meta-analysis.

### Publication bias

When conducting a meta-analysis with more than ten studies, a funnel plot was created in Stata 17 to evaluate the presence of publication bias [25]. To assess the risk of publication bias, the funnel plots were examined, and both Egger’s test and Begg’s test (α = 0.05) were performed. Therefore, in the present study, we assessed publication bias in our primary outcomes (first-degree perineal laceration and second-degree perineal laceration), which were reported in 10 studies [26, 27]. P value < 0.05 was considered statistically significant.

## Results

### Results of the literature search and characteristics of included studies

**Fig. 1:**
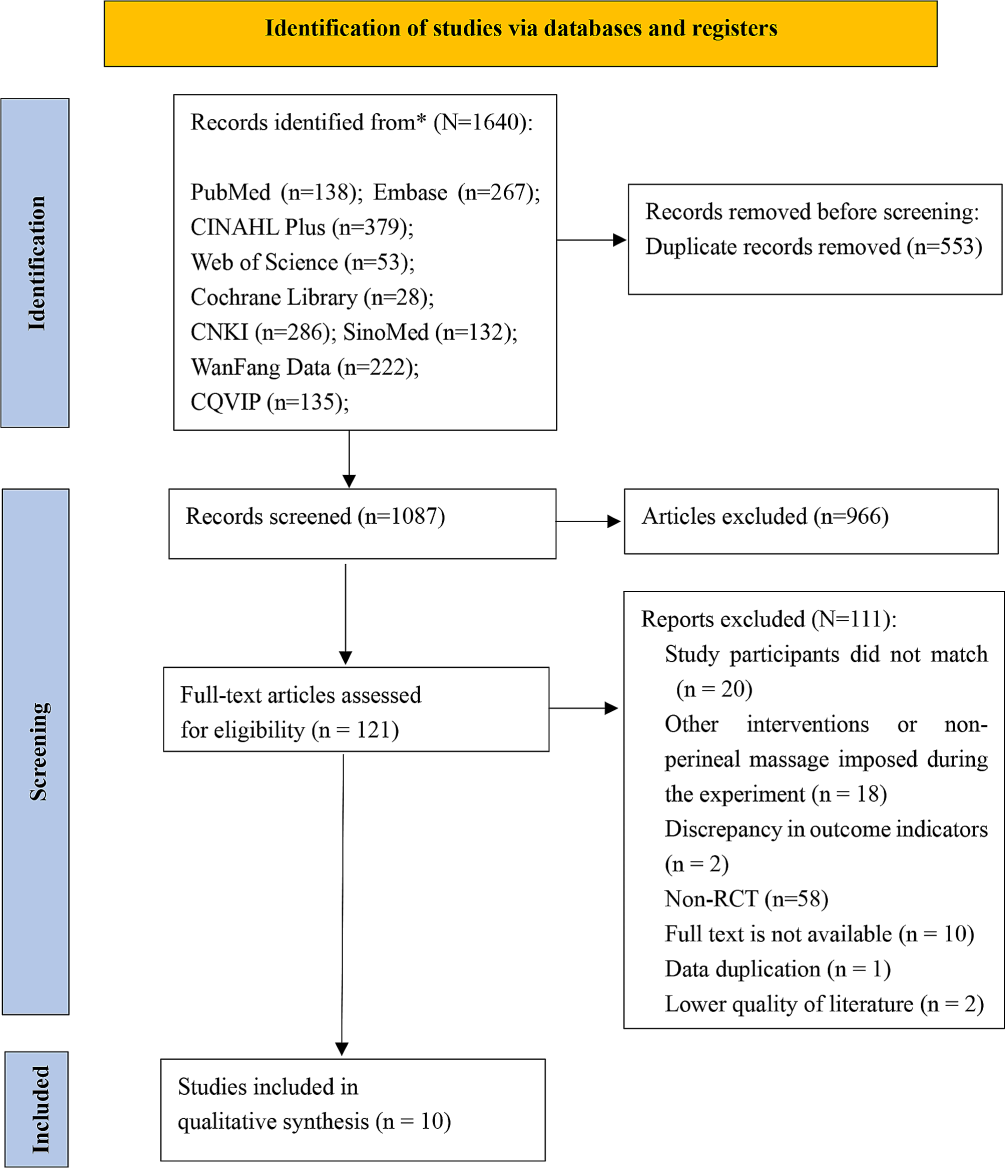
**PRISMA Flow chart of search and study inclusion process**

The literature search initially turned up a total of 1640 items. Of these, 865 were written in English and 775 in Chinese. 1087 articles were left after 553 duplicate entries were eliminated. 1077 papers that didn’t fit the inclusion criteria were eliminated after the titles, abstracts, and full texts were reviewed. Ultimately, ten articles [1, 16–18, 27–32]—six in Chinese [16, 27–31] and four in English [1, 17, 18, 32]—were included in the review. Seven studies [16, 17, 27–31] concentrated on perineal massage beginning in the second stage of labor, whereas three articles [1, 18, 32] concentrated on perineal massage beginning in the antenatal period. The flowchart of the literature selection process is shown in Fig. 1. A total of 10 included studies with a combined sample size of 1,057 participants were identified. The intervention group consisted of 530 participants, while the control group consisted of 527 participants. The basic characteristics of the included studies are presented in Table 1.

### Risk of bias assessment

The quality of included RCTs ranged from low to moderate quality based on the Cochrane risk of bias 2.0 assessment tool. The summary of risk of bias assessment for the included RCTs is shown in supplementary fig. no.2.

### Outcomes

#### Intact perineum

**Fig. 2 Forest plot of the effect of perineal massage during late pregnancy and second stage of labor on the occurrence of intact perineum:**
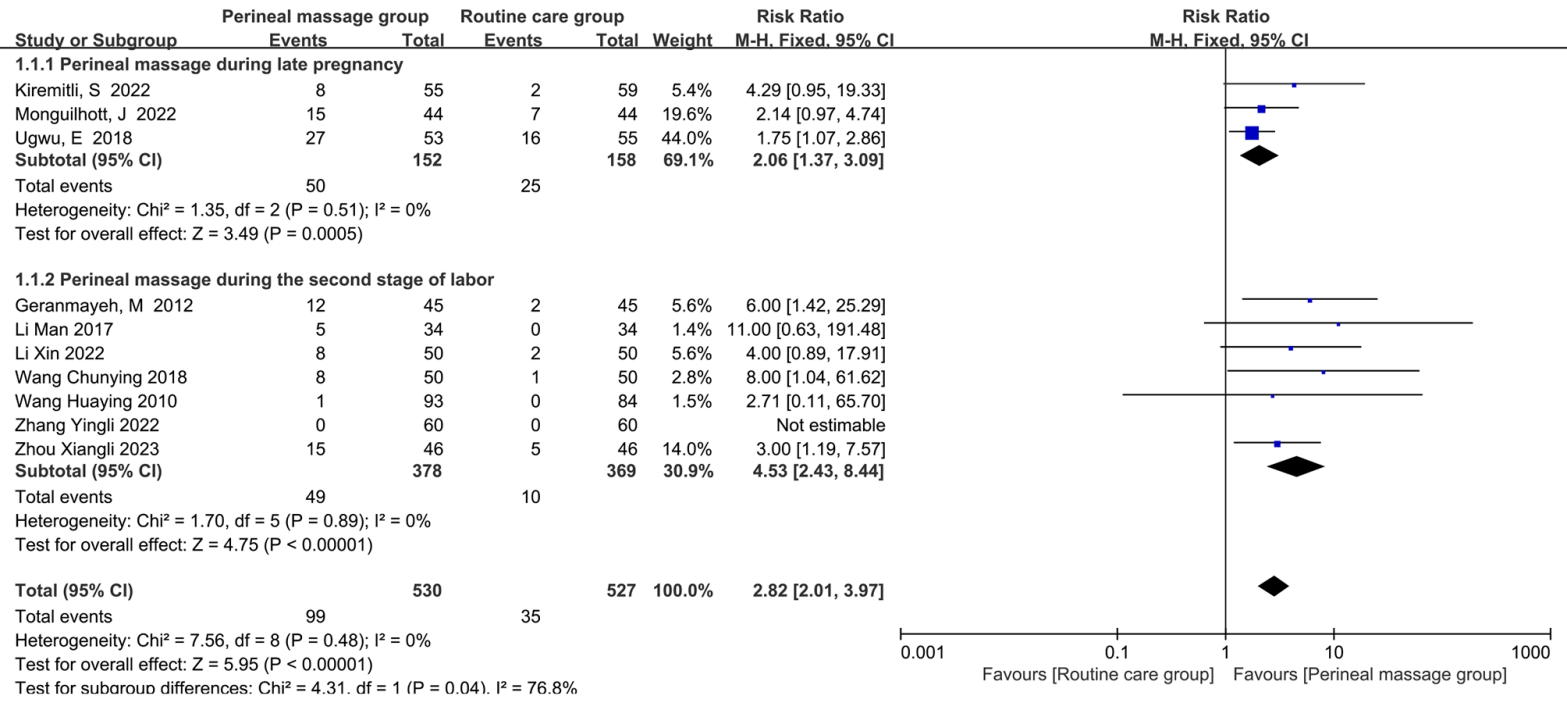
.

There were 10 studies that reported on the rates of perineal integrity. Since perineal integrity rate was a dichotomous variable and I^2^ = 0% suggested that there was no heterogeneity in the studies, the combined effect size was shown using RR and fixed-effects models. The pooled results showed a significant increase in perineal intactness following perineal massage (RR = 2.82, 95% CI: [2.01, 3.97], *P* < 0.001, Fig. 2).

### Perineal lacerations

#### First-degree perineal laceration

**Fig. 3 Forest plot of the effect of perineal massage during late pregnancy and second stage of labor on first-degree perineal laceration:**
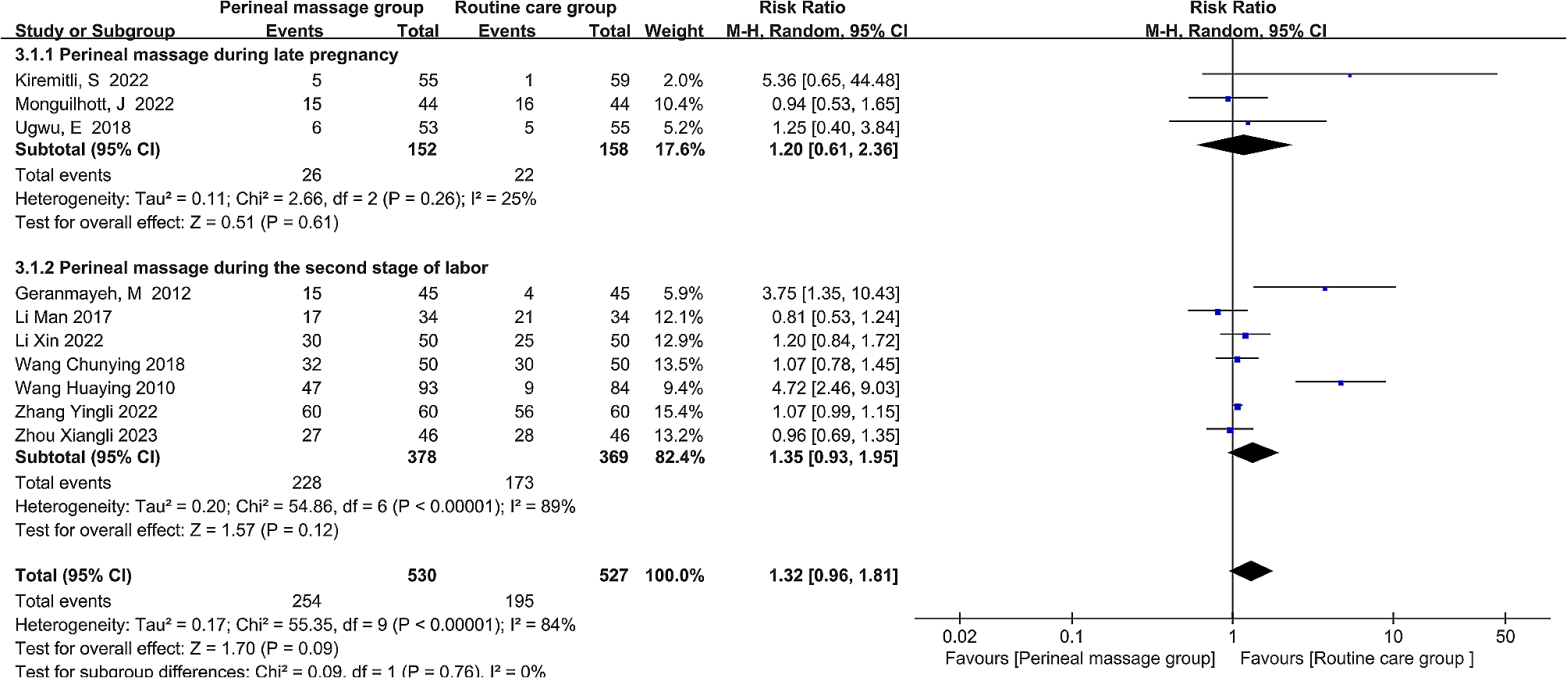
.

The incidence of first-degree perineal lacerations was documented in ten different investigations. Based on the binary outcome of first-degree perineal lacerations and the heterogeneity across the studies, as shown by I^2^ = 84%, the risk ratio (RR) and random-effects model were utilized to calculate the pooled effect size. The rate of first-degree perineal lacerations after receiving the perineal massage did not significantly change, according to the overall data (RR = 1.32, 95% CI: [0.96, 1.81], *P* = 0.09, Fig. 3). Sensitivity analysis showed that the Huaying Wang et al. study [30] was the cause of heterogeneity for first-degree perineal lacerations by progressively removing individual studies. Following its removal, the heterogeneity between the remaining trials was deemed acceptable (I^2^ = 34%, *P* = 0.14). The meta-analysis employed a fixed-effects model. Consistent with the initial findings, the results demonstrated that perineal massage administered during antenatal and the second stage of labor did not significantly affect the rate of first-degree perineal lacerations (RR = 1.07, 95% CI: [0.92, 1.25], *P* = 0.38).

### Second-degree perineal laceration

**Fig. 4 Forest plot of the effect of perineal massage during late pregnancy and second stage of labor on second-degree perineal laceration:**
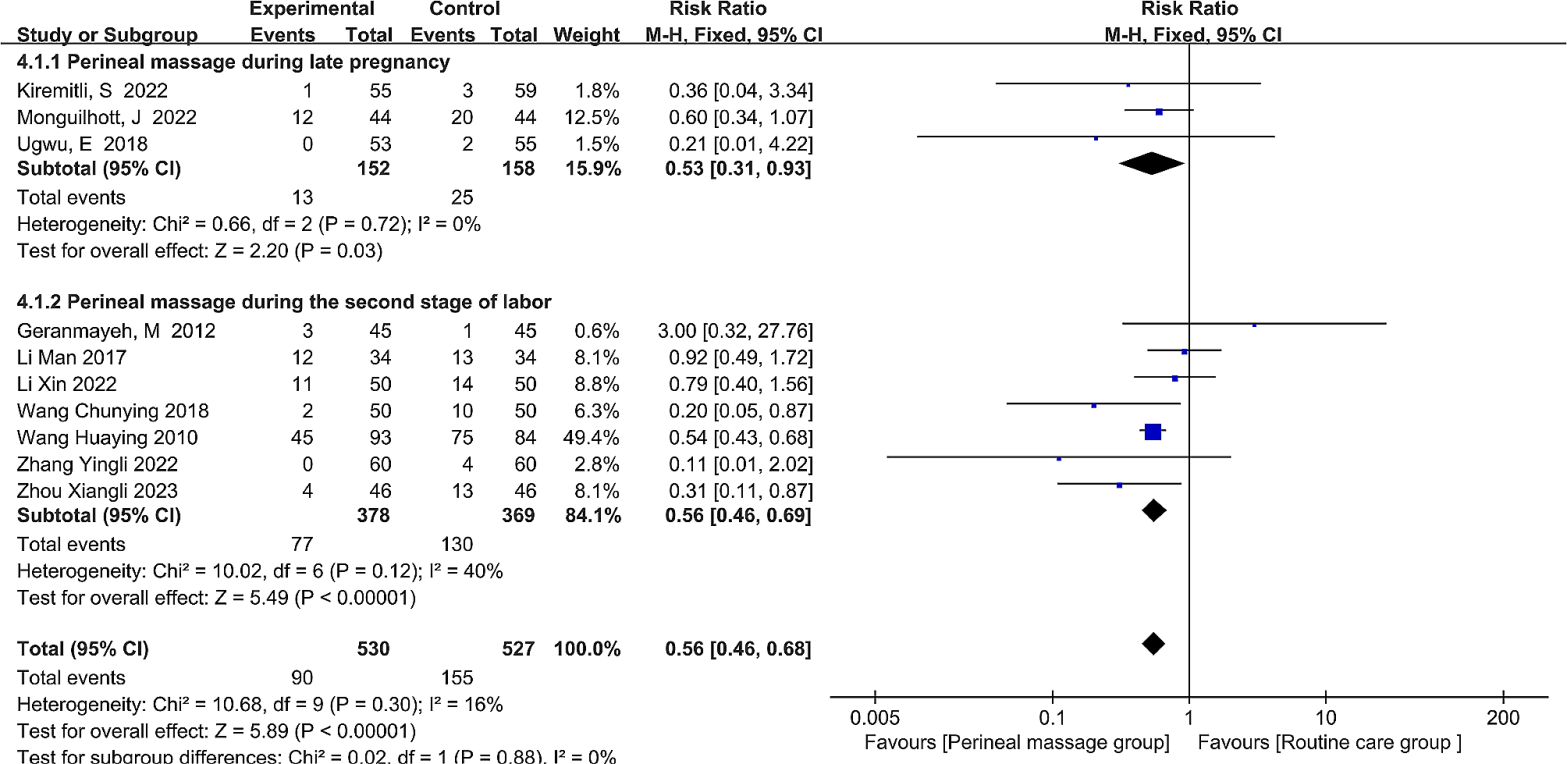
.

Ten distinct studies recorded the incidence of second-degree perineal lacerations. With an I^2^ of 16%, the rate of second-degree perineal laceration was a dichotomous variable. RR and fixed-effects models were used to depict the overall effect size. The combined data showed a significant reduction in the rate of second-degree perineal lacerations after receiving perineal massage (RR = 0.56, 95% CI: [0.46, 0.68], *P* < 0.001, Fig. 4).

### Third- and fourth-degree perineal lacerations

On the frequency of third-degree perineal tears, just one study was conducted. A third-degree perineal tear was experienced by one individual in the perineal massage group and seven participants in the control group in this investigation [18]. Among primiparous women, there were no reports of fourth-degree perineal tears. Nevertheless, it is not feasible to produce a forest plot for visualization because there is only one study accessible.

### Incidence of episiotomy

**Fig. 5 Forest plot of the effect of perineal massage during late pregnancy and second stage of labor on the incidence of episiotomy:**
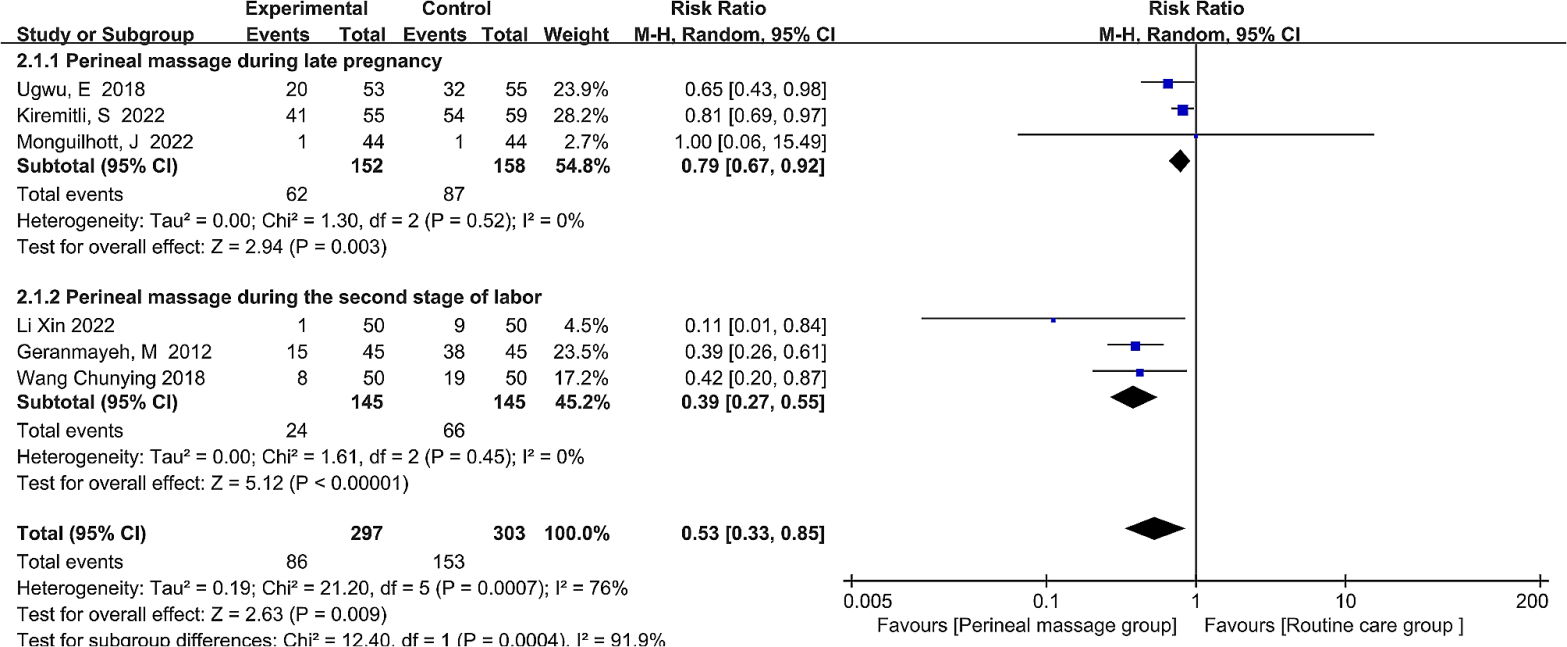
.

The rate of episiotomy was reported in a total of 6 investigations. With the rate of episiotomy being a binary outcome and I^2^ = 76% suggesting heterogeneity between the studies, the pooled effect size was estimated using the random-effects model and risk ratio (RR). The rate of episiotomy after receiving the perineal massage was significantly lower, according to the overall data (RR = 0.53, 95% CI: [0.33, 0.85], *P* = 0.009, Fig. 5). Sensitivity analysis using a sequential exclusion of specific studies showed that the source of the variation in episiotomy rates was the study of Kiremitli, S [18] et al. Following its removal, the heterogeneity between the remaining trials was deemed acceptable (I^2^ = 26%, *P* = 0.25). The meta-analysis employed a fixed-effects model. Consistent with our initial findings, the results demonstrated that perineal massage administered during antenatal and the second stage of labor had a statistically significant impact on reducing the rate of episiotomy (RR = 0.46, 95% CI: [0.35, 0.61], *P* < 0.001).

### Duration of the second stage of labor

The duration of the second stage of labor was the subject of six investigations. Standardized mean difference (SMD) and random-effects model were employed to estimate the pooled effect size because the second stage duration is a continuous variable that is reported in multiple units (hours or minutes) and the I^2^ value is 97%, indicating substantial heterogeneity among the studies. Overall, the results demonstrated that perineal massage significantly shortened the duration of the second stage of labor (SMD = -1.85, 95% CI: [-2.94, -0.76], *P* < 0.001, Fig. 6). Sensitivity analysis was done to look at the effect size’s stability by removing studies one after the other [33]. The robustness and dependability of the analysis’s findings are demonstrated by the observed effect size, which stayed within the overall effect’s confidence interval.

**Fig. 6 Forest plot of the effect of perineal massage during late pregnancy and second stage of labor on the duration of the second stage of labor:**
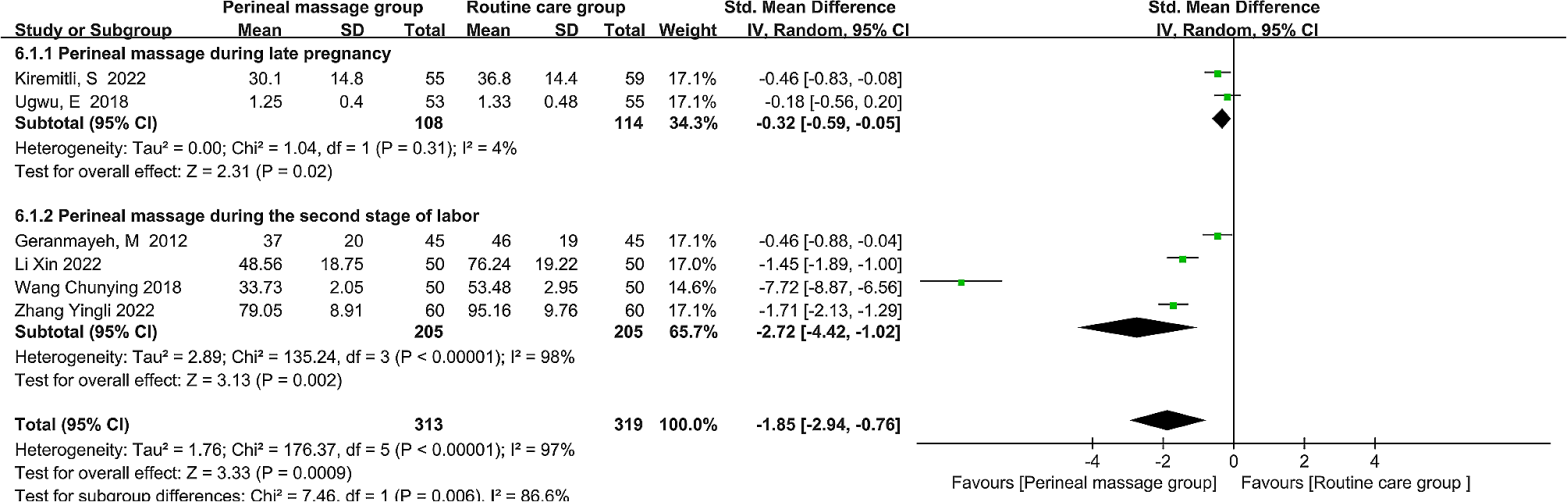
.

### Postpartum hemorrhage

Only perineal massage at the beginning of the second stage of labor reported postpartum hemorrhage in five studies. With postpartum hemorrhage being a continuous variable and I^2^ = 97% signifying study heterogeneity, the combined effect size was determined using random effects and MD models. Following perineal massage, postpartum hemorrhage significantly decreased (MD = -59.46, 95% CI: [-80.79, -38.12], *P* < 0.001, supplementary Fig. no.3). After excluding each included study individually, the sensitivity analysis revealed that the heterogeneity in postpartum hemorrhage originated from Chunying Wang et al. study [29]. The heterogeneity between the remaining studies was deemed acceptable (I^2^ = 21%, *P* = 0.28) following their exclusion. After selecting a fixed-effects model for the meta-analysis, it was found that, in line with the initial findings, perineal massage at the start of the second stage of labor had a statistically significant impact on postpartum hemorrhage (MD = -50.97, 95% CI [-55.80, -46.14], *P* < 0.001).

### Perineal pain

**Fig. 7 Forest plot of the effect of perineal massage during late pregnancy and second stage of labor on perineal pain:**
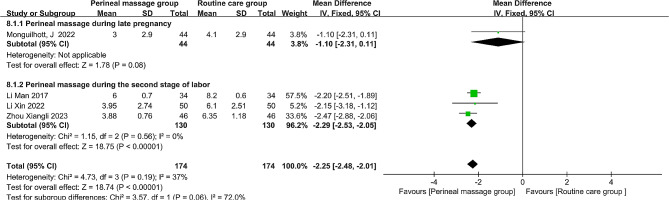
.

A total of five investigations, including one that involved perineal massage at the start of the second stage of labor, documented recent postpartum perineal pain in primiparous women [31]. In this study, the prevalence of each level of perineal pain was measured on a scale ranging from 0 to 3 [31]. This type of data differs from other studies that have used perineal pain as an outcome indicator because it is incomplete and cannot be converted into numerical data. Therefore, this study was not included in the forest mapping analysis. MD and fixed-effects models were employed to show the combined effect size because the data on recent postpartum perineal pain was a continuous variable with an I^2^ of 37%, indicating that there was no heterogeneity in the study. The aggregated results showed a substantial decrease in recent perineal pain following perineal massage (MD = -2.25, 95% CI: [-2.48, -2.01], *P* < 0.001, Fig. 7). Noticeably, perineal massage given to primiparous women during the antenatal period had no significant effect on recent postpartum pain (MD= -1.10, 95% CI: [-2.31, 0.11], *P* = 0.08, Fig. 7). On the contrary, perineal massage given during the second stage of labor had a significant effect (MD= -2.29, 95% CI: [-2.53, -2.05], *P* < 0.001, Fig. 7).

### Urinary incontinence

**Fig. 8 Forest plot of the effect of perineal massage during late pregnancy on urinary incontinence:**
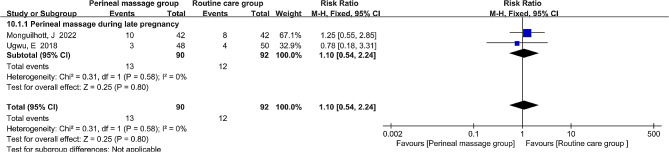
.

Just two literatures measured the effects of antenatal perineal massage on postpartum urinary incontinence in primiparous women at three months postpartum. A risk ratio (RR) and fixed-effects model were utilized to assess the pooled effect size because urine incontinence is a binary variable and the I^2^ value is 0%, indicating no heterogeneity among the studies. Perineal massage did not significantly reduce urine incontinence, according to the results (RR = 1.10, 95% CI: [0.54, 2.24], *P* = 0.80, Fig. 8).

### Fecal incontinence

**Fig. 9 Forest plot of the effect of perineal massage during late pregnancy on fecal incontinence:**
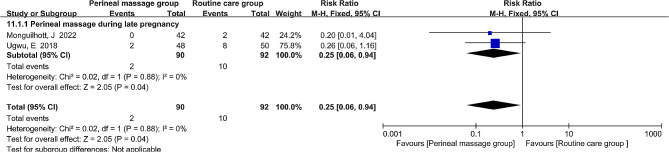
.

Just two literatures measured the effects of antenatal perineal massage on postpartum fecal incontinence in primiparous women at three months postpartum. RR and fixed-effect models were utilized to depict the combined effect size because fecal incontinence is a binary variable with an I^2^ = 0%. Perineal massage was found to reduce the incidence of fecal incontinence (RR = 0.25, 95% CI: [0.06, 0.94], *P* = 0.04, Fig. 9).

### Flatus incontinence

**Fig. 10 Forest plot of the effect of perineal massage during late pregnancy on flatus incontinence:**
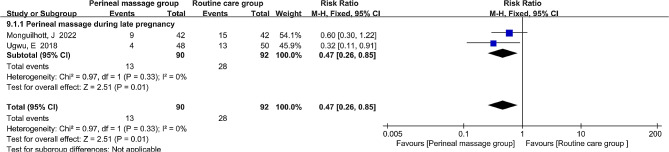
.

Just two literatures measured the effects of antenatal perineal massage on postpartum flatus incontinence in primiparous women at three months postpartum. As flatus incontinence is a binary variable with an I^2^ value of 0%, suggesting no heterogeneity across the trials, the pooled effect size was estimated using a risk ratio (RR) and fixed-effects model. Perineal massage was found to reduce the incidence of flatus incontinence (RR = 0.47, 95% CI: [0.26, 0.85], *P* = 0.01, Fig. 10).

### Publication bias

Using Stata 17 to create a funnel plot and observe publication bias. Ten articles on intact perineum, first-degree perineal tear, and second-degree perineal tear are included in this review. One study that was included in the category of intact perineum found zero incidence rates for both the control and experimental groups [31]. Using RevMan 5.3, this specific study is automatically removed when creating a forest plot. Consequently, a funnel plot for this result cannot be produced because there are nine investigations on intact perineum that are still ongoing. Supplementary Fig. no. 4 displays the funnel plot of first-degree perineal tear with publication bias test results, and supplementary Fig. no. 5 displays the funnel plot of second-degree perineal tear with publication bias test results. Both funnel plots appear to have a symmetrical, uniformly dispersed shape upon visual inspection, suggesting a low likelihood of publishing bias. The findings of the Begg’s test (*P* = 0.107) and Egger’s test (*P* = 0.189) publication bias tests for first-degree perineal tears are as follows: *P* > 0.05 is shown in both cases, and the 95% confidence interval, which covers the range from − 0.630 to 2.707 (including 0), implies that the influence of publication bias on the outcomes is minimal. The outcomes of the publication bias tests for a second-degree perineal tear are as follows: *P* = 0.683 for Egger’s test and *P* = 0.592 for Begg’s test. *P* > 0.05 is shown in both cases, and the 95% confidence interval, which covers the range from − 1.423 to 0.982 (including 0), suggests that publication bias had little effect on the outcomes.

## Discussion

This systematic review and meta-analysis presented herein contribute to the existing literature by reinforcing the advantages of perineal massage for primiparous women, particularly during the antenatal period and the second stage of labor. Our findings are in concordance with previous studies, which have established the benefits of perineal massage. Notably, our analysis supports the notion that perineal massage administered during the second stage of labor is associated with a potentially more effective reduction in early postpartum perineal pain. On the other hand, there are a number of outcomes pertaining to the perineum that can be improved by perineal massage throughout the antenatal period and the second stage of labor, including increased perineal integrity, decreased incidence of episiotomy, and decreased rates of second-degree perineal tears. However, neither perineal massage timing was associated with a statistically significant decrease in the occurrence of first-degree perineal tears. During the antenatal and the second stage of labor, perineal massage can help reduce the duration of the second stage of labor. Only perineal massage during antenatal reported significant effects on fecal incontinence and flatus incontinence at three months postpartum, while its effect on urinary incontinence was not significant. Furthermore, only perineal massage during the second stage of labor reported a reduction in postpartum hemorrhage.

The results of the systematic review indicate that perineal massage performed during the second stage of labor may be more effective in reducing early postpartum perineal pain (during immediately after delivery) in primiparous women compared to perineal massage performed in antenatal, which is consistent with the findings of Milka et al. [34]. This may be attributed to the potential of perineal massage to alleviate the burning sensation and perineal pain during labor [35]. However, these findings contradict previous reviews. Seehusen et al. found that perineal massage did not have a significant effect on short-term relief of perineal pain, but it showed a relatively significant effect in relieving long-term perineal pain (at 3 months postpartum) [36]. Nevertheless, other studies have indicated that perineal massage can alleviate immediate and 3-month postpartum perineal pain [37]. Due to the limited number of studies included in this review, the results may not be sufficiently stable. Therefore, further research is encouraged to explore the effects of perineal massage on reducing the duration of perineal pain.

According to the findings of the systematic review, women’s perineal outcomes can be significantly improved by perineal massage administered throughout the antenatal period and the second stage of labor. The results of earlier systematic reviews on perineal massage during the antenatal and the second stage of labor [38, 39] are in line with this. This could be because the full dilatation of the cervix and the fetus’s delivery, which normally take 1–2 h for primiparous women, are the two main components of the second stage of labor [40]. The baby’s head and body stay in the birth canal for a long time during this procedure, applying pressure to the perineum and surrounding tissues repeatedly and for a long time, increasing the risk of tearing. In order to mimic the drop of the baby’s head, the fingers might provide downward pressure inside the vagina during perineal massage [38]. Additionally, perineal massage increases the rate of perineal integrity by lessening the fetus’s susceptibility to excessive pressure or mechanical tension during its passage through the birth canal and improving the extensibility, elasticity, and strength of the perineal muscles [39]. It also lowers the risk of perineal lacerations and episiotomies. Based on systematic rating data, perineal massage may be helpful in reducing second-degree perineal lacerations. Women should be made aware of perineal massage as a means of reducing perineal trauma, which can lessen the likelihood of second- and higher-degree perineal injuries, according to the Queensland Clinical Guidelines [41]. Furthermore, the perineum is more easily dilated and stretched during fetal passage because of the enhanced stretchability and suppleness of the perineal tissues, as well as the increased flexibility and responsiveness of the perineal muscles as a result of perineal massage. This shortens the time of the second stage of labor by reducing resistance and friction and aiding the baby’s descent and birth.

Perineal massage during antenatal has a significant effect on fecal incontinence and anal gas incontinence at three months postpartum, but its effect on urinary incontinence is not clear. Perineal massage can help to increase the elasticity and flexibility of the perineal muscles. Furthermore, perineal massage’s biomechanical action can widen blood vessels in the perineal area, boosting blood flow, enhancing local circulation, and encouraging the development of perineal muscles to guard against perineal injuries [42, 43]. By decreasing perineal injuries, this may assist to improve the long-term impact of perineal injuries. Finally, it can strengthen the pelvic floor muscles during the postpartum period, which is beneficial for pelvic floor dysfunction disorder and thereby reduce postpartum fecal and flatus incontinence. Future research should include more rigorous randomized controlled trials to further investigate the effects of perineal massage on urinary incontinence and extend the follow-up period.

The systematic review’s findings imply that perineal massage may be beneficial for postpartum hemorrhage. That could be because it makes the perineal muscles more elastic and flexible, which makes the perineal region more extensible during labor and lessens the need for perineal tissue lacerations and episiotomies as well as the risk of postpartum hemorrhage [26]. By strengthening and lengthening the perineal muscles, perineal massage lowers the risk of hemorrhage by enabling the perineal region to support and stabilize the birthing process with more energy.

This review is limited by the small number of included studies and some observed heterogeneity in the outcomes. The primary causes of the research’s variability were variations in the perineal massage technique, length, number of massages, and operator skill. To validate our study’s findings, more large-scale, multicenter randomized controlled studies are required. Future research is required to evaluate the effects of various massage frequencies and durations, as well as the dosage of perineal massage, on pregnancy. Further research on the long-term effects of perineal massage is also worthwhile because it may improve women’s quality of life and postpartum recovery. In order to find the evidence as thoroughly as possible, we finally devised a methodical and thorough search strategy. Nonetheless, the limits of the Chinese and English language types may result in publishing bias and encourage future research to be conducted on other language literature.

## Conclusion

According to the results of this review and meta-analysis, perineal massage, both antenatally and during the second stage of labor, is effective in reducing perineal injury, and it is particularly effective in reducing perineal pain in the early postpartum period when performed at the beginning of the second stage of labor. Although the effect of perineal massage on urinary incontinence is uncertain, it has been demonstrated that antenatal perineal massage significantly reduces the incidence of fecal and flatus incontinence at three months postpartum. However, there are no studies specifically addressing the effect of perineal massage during the second stage of labor on improving pelvic floor function in the early postpartum period. This study suggests that if perineal massage is not administered during the antenatal phase, it is feasible to preserve perineal integrity and reduce perineal injuries and postpartum hemorrhage by administering perineal massage during the second stage of labor. It would be beneficial to support more thorough randomized controlled trials in the future to investigate the effects of perineal massage at different periods as well as the mother’s long-term outcomes.

**Table 1:** Characteristics of the included studies (*N* = 10)

Authors/	Mean	Country	Size	Intervention								Comparison	Outcome
Years	Age of participants												
	(I/C)		(I/C)	Performer	Start time	Technique	Specific operation time	Frequency	Stop time	Total duration	Auxiliary		
Li, 2017 [27]	28.4 ± 2.1/	China	68 (34/34)	Midwife	Second stage of labor	Massage the index and middle fingers on the perineum.	During contractions	Massage each contraction about 4 times.	The fetal head is crowned.	Not provided	Lubricant (Sterile peanut oil)	Routine care	(1) Intact perineum
	28.3 ± 2.1												(2) First-degree perineal laceration
													(3) Second-degree perineal laceration
													(4) Duration of the second stage of labor
													(5) Perineal pain
Li, 2022	28.01 ± 3.50/	China	100	Midwife	Second stage of labor	Insert the middle finger and index finger into the vagina, about 2-3cm in size, and massage the inverted U from 3 o’clock to 9 o’clock.	During contractions	Not provided	When the head of the fetus is 2–3 cm crowned.	Not provided	Lubricant	Routine care	(1) Intact perineum
[28]	28.63 ± 3.27		(50/50)								(Sterile liquid paraffin wax)		(2) First-degree perineal laceration
													(3) Second-degree perineal laceration
													(4) Incidence of episiotomy
													(5) Duration of the second stage of labor
													(6) Postpartum hemorrhage
													(7) Perineal pain
Wang,	28.1 ± 3.6/	China	100	Midwife	Second stage of labor	Put the right index finger and middle finger into the perineum until the second joint of the fingers, and proceed clockwise with the circular massage.	During contractions	Massage 3 to 5 times during contractions.	The fetal head is crowned.	Not provided	Lubricant	Routine care	(1) Intact perineum
2018	27.7 ± 3.5		(50/50)								(Silicone oil cotton balls)		(2) First-degree perineal laceration
[29]													(3) Second-degree perineal laceration
													(4) Incidence of episiotomy
													(5) Postpartum hemorrhage
													(6) Perineal pain
Wang,	28.07 ± 3.05/	China	177	Midwife	Second stage of labor	The right index finger and middle finger are placed in the perineal body part clockwise from 3 to 9 o’clock for circular massage.	During contractions	Massage 3 to 6 times during contractions.	The fetal head is crowned.	Not provided	Lubricant	Routine care	(1) Intact perineum
2010	28.36 ± 3.03		(93/84)								(Sterile peanut oil)		(2) First-degree perineal laceration
[30]													(3) Second-degree perineal laceration
													(4) Duration of the second stage of labor
													(5) Perineal pain
Zhang, 2022	26.83 ± 3.17/	China	120	Midwife	Second stage of labor	The right index finger and middle finger are placed in the perineum at 3–9 o’clock for a clockwise massage with even force.	Between contractions	Not provided	The fetal head is crowned.	Not provided	Lubricant	Routine care	(1) Intact perineum
[31]	26.17 ± 3.95		(60/60)								(Sterile liquid paraffin wax)		(2) First-degree perineal laceration
													(3) Second-degree perineal laceration
													(4) Duration of the second stage of labor
													(5) Postpartum hemorrhage
													(6) Perineal pain
Geranmayeh, M et al.,	21 ± 3/	Tehran, Iran	90	Midwife	Second stage of labor	Not provided.	Uterine contractions	Not provided	The fetal head is crowned.	Not provided	Lubricant	Routine care	(1) Intact perineum
2012	22 ± 3		(45/45)								(Vaseline)		(2) First-degree perineal laceration
[17]													(3) Second-degree perineal laceration
													(4) Incidence of episiotomy
													(5) Postpartum hemorrhage
Zhou,	26.85 ± 2.09/	China	92	Midwife	Second stage of labor	The right index finger and middle finger are extended into the maternal vagina; the depth needs to be within 3cm, and then the "U"-shaped track is followed by clockwise massage.	During contractions	Not provided	The fetal head is crowned.	Not provided	Lubricant	Routine care	(1) Intact perineum
2023	26.92 ± 2.14		(46/46)								(Sterile liquid paraffin wax)		(2) First-degree perineal laceration
[16]													(3) Second-degree perineal laceration
													(6) Duration of the second stage of labor
Kiremit-li, S et al.,2012	25.9 ± 4.1/	Turkey	114	Midwife	After 34 weeks	Placing one or two fingers (preferably the index and middle fingers) 3–4 cm inside the vagina and stretching them towards the rectum in a U shape from 3 o’clock to 9 o’clock.	No contraction	Not provided	Until the day of delivery	10 minutes a day	Lubricant	Not provided	(1) Intact perineum
[18]	24.8 ± 3.6		(55/59)								(olive oil)		(2) First-degree perineal laceration
													(3) Second-degree perineal laceration
													(4) Third-degree perineal lacerations
													(5) Incidence of episiotomy
													(6) Postpartum hemorrhage
Mongui-lhott,S et al.,2022 [32]	29.5 ± 5.5/	Brazilian	88	pregnant woman and/or companion	34 weeks of pregnancy	Introducing one or two fingers three to four centimeters deep into the entrance of the vagina, first applying and maintaining downward pressure for two minutes and then for another two minutes on each side.	No contraction	Perineal massage for at least 5 minutes in each session for at least 10 days.	Until the day of delivery	daily for 5–10 minutes	Lubricant	Usual antenatal care	(1) Intact perineum
	31.1 ± 6.1		(44/44)								(almond oil)		(2) First-degree perineal laceration
													(3) Second-degree perineal laceration
													(4) Incidence of episiotomy
													(5) Duration of the second stage of labor
													(6) Urinary incontinence
													(7) Fecal incontinence
													(8) Flatus incontinence
Ugwu, E et al.,2018[1]	28.02 ± 4.35/	Nigeria	108	pregnant woman herself or by her husband	34–36 weeks	Inserting the thumb and index finger, 3–5 cm into the vagina.	None.	Not provided	Until the day of delivery	perform 10 min of daily	Lubricant	Routine care	(1) Intact perineum
	28.77 ± 3.70		(53/55)								(KY jelly)		(2) First-degree perineal laceration
													(3) Second-degree perineal laceration
													(4) Incidence of episiotomy
													(5) Postpartum hemorrhage
													(6) Urinary incontinence
													(7) Fecal incontinence
													(8) Flatus incontinence

### Electronic supplementary material

Below is the link to the electronic supplementary material.

Supplementary Material 1

Supplementary Material 2

Supplementary Material 3

Supplementary Material 4

Supplementary Material 5

## Data Availability

All data used in this meta‑analysis are included in this article and its supplementary materials or are publicly available from the original sources.

## Abbreviations

RCTs: Randomized controlled trials
PFMT: Pelvic floor muscle training
PRISMA: Preferred Reporting Item for Systematic Reviews and Meta-analyses
PICOS: Population, Intervention, Comparison, Outcome, Study Design
CNKI: China National Knowledge Infrastructure
CQVIP: China Science and Technology Journal Database
VAS: Visual analog scale
MD: Mean difference
SMD: Standardized mean difference
RR: Relative risks
CI: Confidence interval
