# Supplementary material for: Effects of perineal massage at different stages on perineal and postpartum pelvic floor function in primiparous women: a systematic review and meta-analysis
Source: BMC Pregnancy Childbirth. 2024 Jun 3;24:405. doi: 10.1186/s12884-024-06586-w (PMC11149294; doi:10.1186/s12884-024-06586-w)
Supplement: Supplementary file 1 — Supplementary Material 1 [file 12884_2024_6586_MOESM1_ESM.docx]

**Supplementary file no.1 PICOS and Search strategy**

**Search terms used in PICOS search**

| PICOS | Definition | Search keywords |
| --- | --- | --- |
| Population | Primiparous women | Not set |
| Intervention | Perineal massage | “Perine*” AND（ “Massage” OR “Zone Therapy” OR “Therapies, Zone” OR “Zone Therapies” OR “Therapy, Zone” OR “Massage Therapy” OR “Massage Therapies” OR “Therapies, Massage” OR “Therapy, Massage”） |
| Comparison | Treatment as usual | Not set |
| Outcomes | perineal integrity, perineal injury, perineal pain, duration of the second stage of labor, postpartum hemorrhage, urinary incontinence, fecal incontinence, and flatus incontinence. | Not set |
| Study design | RCTs | Not set |

**Search Strategies in PubMed and China National Knowledge Infrastructure**

| Database | Numerical order | Search terms and strategies |
| --- | --- | --- |
| PubMed  CNKI | #1  #2  #3  #4  #5  #6  #7  #1  #2  #3 | “Perineum” [Mesh]  Perine*[Title/Abstract]  #1 OR #2  “Massage” [Mesh]  Massage [Title/Abstract] OR “Zone Therapy”  [Title/Abstract] OR “Therapies, Zone” [Title/Abstract] OR “Zone Therapies” [Title/Abstract] OR “Therapy, Zone” [Title/Abstract] OR “Massage Therapy”  [Title/Abstract] OR “Massage Therapies” [Title/Abstract] OR “Therapies, Massage” [Title/Abstract] OR “Therapy, Massage” [Title/Abstract]  #4 OR #5  #3 AND #6  SU = “会阴”  SU = “按摩”  #1 AND #2 |
