# Supplementary figures and images for: Effects of perineal massage at different stages on perineal and postpartum pelvic floor function in primiparous women: a systematic review and meta-analysis
Source: BMC Pregnancy Childbirth. 2024 Jun 3;24:405. doi: 10.1186/s12884-024-06586-w (PMC11149294; doi:10.1186/s12884-024-06586-w)

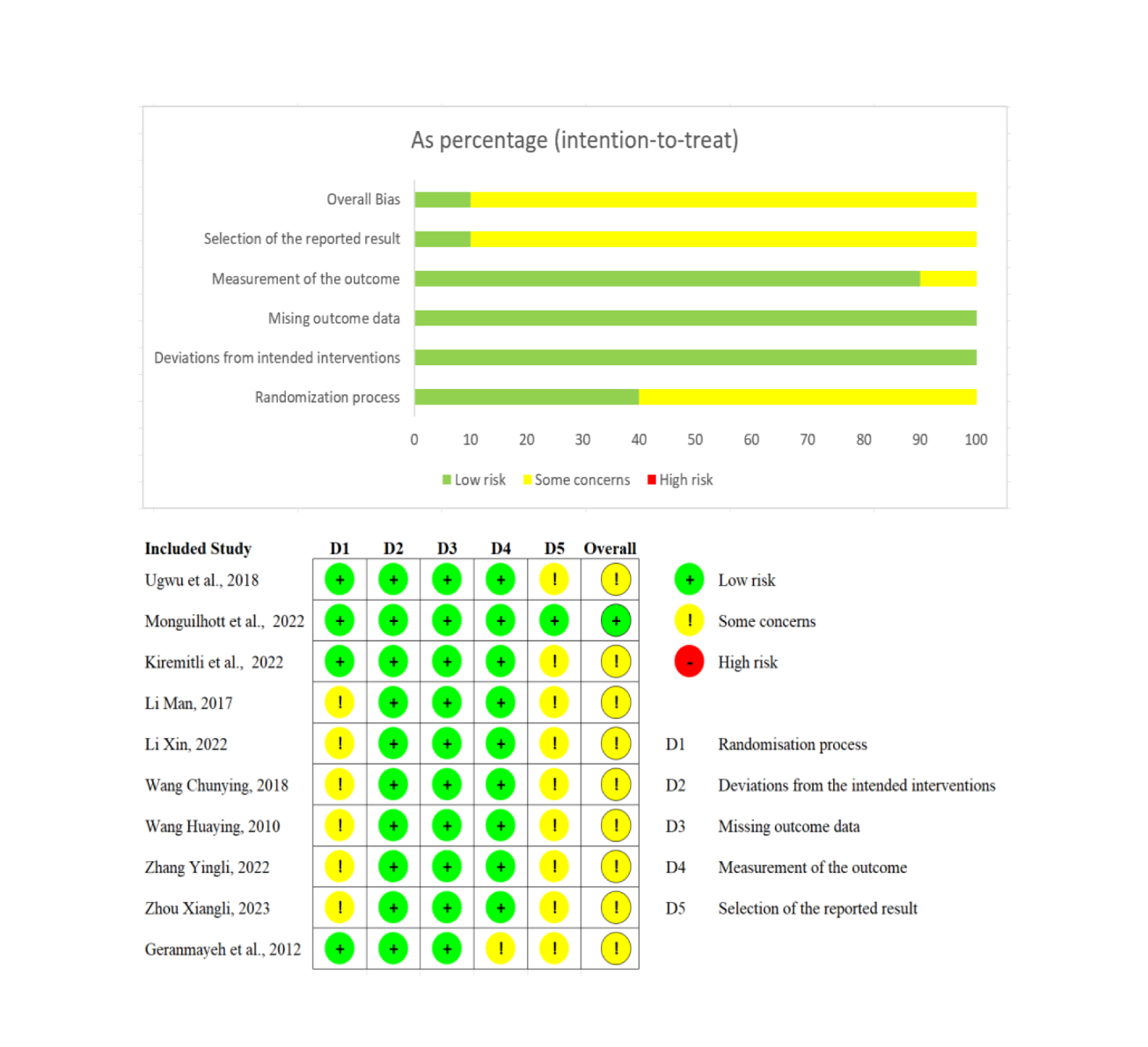

Supplement: Supplementary file 2 — Supplementary Material 2 [file 12884_2024_6586_MOESM2_ESM.docx]

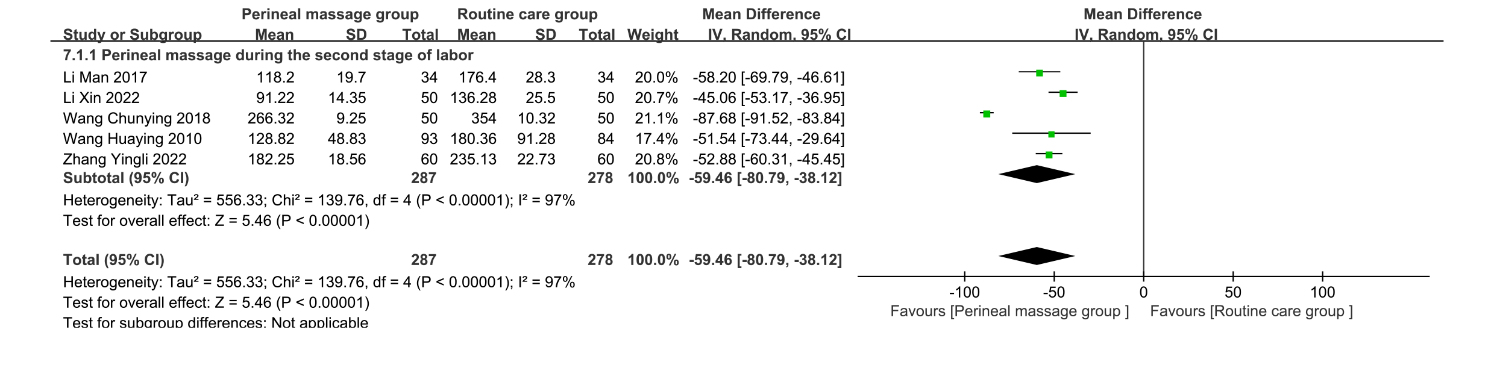

Supplement: Supplementary file 3 — Supplementary Material 3 [file 12884_2024_6586_MOESM3_ESM.docx]

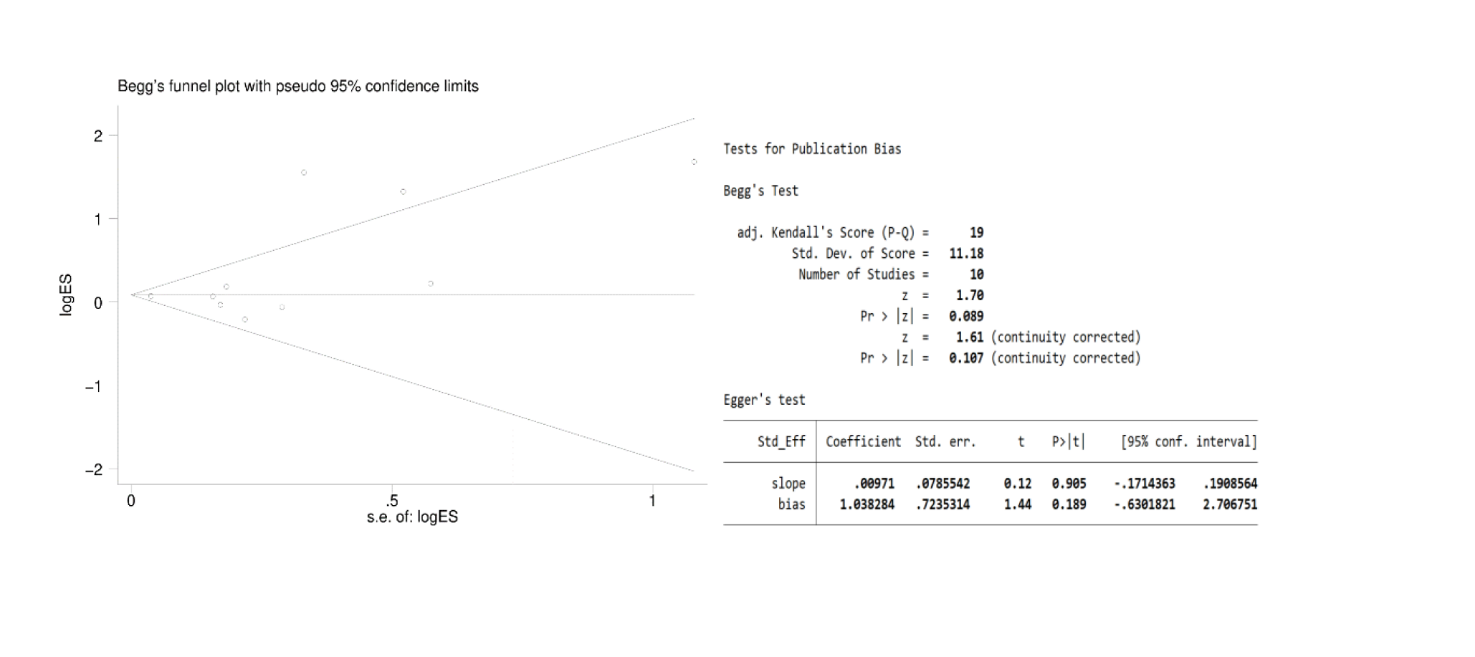

Supplement: Supplementary file 4 — Supplementary Material 4 [file 12884_2024_6586_MOESM4_ESM.docx]

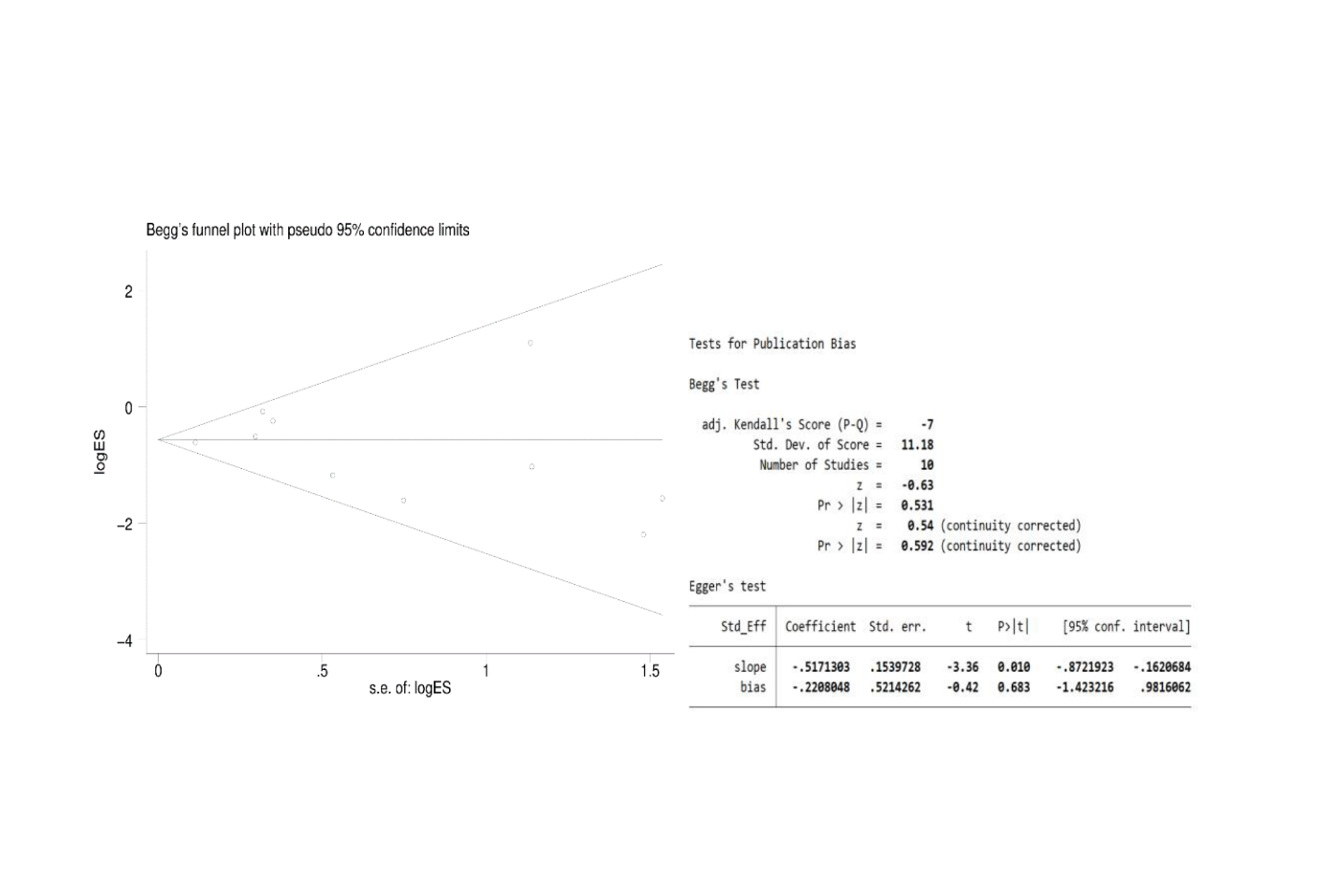

Supplement: Supplementary file 5 — Supplementary Material 5 [file 12884_2024_6586_MOESM5_ESM.docx]
